# Real-world usage, effectiveness, and microbiological features of ceftazidime-avibactam in clinical practice in China

**DOI:** 10.3389/fcimb.2025.1663392

**Published:** 2026-01-27

**Authors:** Xiaohua Qin, Tianxin Xiang, Xiaoju Zhang, Xuzhu Ma, Weifeng Zhao, Yunsong Yu, Caiyan Zhao, Liang Gao, Lifen Li, Tiantian Wang, Chongjie Pang, Xiaoyu Zhao, Renru Han, Felix Cao, Ming Su, Junchao Lu, Wenjuan Xu, Shan Yin, Danni Lu, Xinyu Yang, Minggui Wang

**Affiliations:** 1 Institute of Antibiotics, Huashan Hospital, Fudan University, Shanghai, China; 2 Department of Infectious Diseases, The First Affiliated Hospital of Nanchang University, Nanchang, China; 3 Department of Pulmonary and Critical Care Medicine, Henan Provincial People’s Hospital, Zhengzhou, China; 4 Department of Infectious Diseases, Beijing Tsinghua Changgung Hospital, Beijing, China; 5 Department of Infectious Diseases, The First Affiliated Hospital of Soochow University, Suzhou, China; 6 Department of Liver Diseases and Infectious Diseases, Sir Run Run Shaw Hospital, Zhejiang University School of Medicine, Hangzhou, China; 7 Department of Infectious Diseases, The Third Hospital of Hebei Medical University, Shijiazhuang, China; 8 Department of Neurosurgery, Shanghai Tenth People’s Hospital, Shanghai, China; 9 Department of Critical Care Medicine, The First Affiliated Hospital of Sun Yat-sen University, Guangzhou, China; 10 Department of Hematology, The Affiliated Hospital of Ningbo University Medical College, Ningbo, China; 11 Department of Infectious Diseases, Tianjin Medical University General Hospital, Heping, China; 12 China Medical, Pfizer Inc, Beijing, China; 13 China Medical, Pfizer Inc, Shanghai, China

**Keywords:** carbapenem resistance, ceftazidime-avibactam, clinical outcomes, microbiological outcomes, real-world evidence

## Abstract

**Background:**

Infections due to multidrug- and extensively drug-resistant bacteria can be difficult to treat and are associated with high mortality and burden of disease. Ceftazidime-avibactam (CVA) is used in patients with multidrug-resistant Gram-negative bacterial infections.

**Objective:**

To describe the real-world usage, effectiveness, and antimicrobial features of CVA in clinical practice in China.

**Methods:**

This multicenter, prospective observational study collected medical record data from adult patients (≥18 years) who had been hospitalized and treated with ≥1 dose of CVA. Clinical and microbiological outcomes were evaluated at the end of treatment, with clinical cure defined as resolution of infection following treatment with CVA.

**Results:**

Data were obtained from 220 adult patients receiving ≥1 dose of CVA in China. Infections indicated for CVA included pneumonia (64.5%), complicated intra-abdominal infection (16.8%) and bloodstream infection (7.3%). *Klebsiella pneumoniae* (129/227 isolates, 56.8%) was the most commonly identified pathogen, followed by *Pseudomonas aeruginosa* (33 isolates, 14.5%). Of 87 K*. pneumoniae* isolates, 76 (87.4%) were meropenem resistant and the same proportion carried cabapenemase genes. Low levels of resistance to CVA were observed across all isolates. Three-quarters of patients received CVA as definitive therapy. Clinical cure at end of treatment was achieved in 66.4% of patients and microbiological success in 69.6% of patients.

**Conclusion:**

Findings from this study provide important real-world data on treatment patterns, microbiological features, and clinical and microbiological outcomes for CVA in routine clinical practice in China.

## Introduction

Antimicrobial resistance is an important global health challenge, with resistance to carbapenems in Gram-negative bacteria increasing significantly worldwide from 619,000 associated and 127,000 attributable deaths in 1990, to 1.03 million associated and 216,000 attributable deaths in 2021 (G. B. D. Antimicrobial Resistance Collaborators, 2024). Increased prevalence of carbapenem-resistant Enterobacteriales has been observed in all regions, including the Asia-Pacific region. The China Antimicrobial Surveillance Network (CHINET) noted increasing rates of carbapenem-resistant *Klebsiella pneumoniae* (CRKP) of 26.0–27.5% between 2018 and 2022, together with decreasing, but still marked, rates of carbapenem-resistant strains of *Pseudomonas aeruginosa* of approximately 20–30% (Yang W. et al., 2023). This follows significant increases in resistance rates of *K. pneumoniae* to imipenem, from 2.9% in 2005 to 22.6% in 2022 according to data from hospitals in some regions of China (Qin et al., 2024). Similarly, the China Antimicrobial Resistance Surveillance System noted an increase in CRKP from 6.4% in 2014 to 10.9% in 2019 in a northern province of China (Wang N. et al., 2022). Across the different Chinese provinces, the prevalence of CRKP ranged from 0.8% to 28.1% in 2022, with an average rate of 10.0% (an increase from 7.4% in 2016) (Qin et al., 2024).

Carbapenems are used as a treatment of last resort for Gram-negative infections (Wong and van Duin, 2017). Therefore, the increasing incidence of carbapenem-resistant organisms producing β-lactam hydrolyzing enzymes (β-lactamases) has resulted in increasing carbapenem resistance, which poses a major therapeutic challenge (Wong and van Duin, 2017; G. B. D. Antimicrobial Resistance Collaborators, 2024). Infections due to Carbapenem-Resistant Enterobacterales (CRE) can be difficult to treat and are associated with high morbidity, mortality, and burden of disease (Salam et al., 2023). The 2024 World Health Organization Bacterial Priority Pathogens List (WHO BPPL), an important tool in the global fight against antimicrobial resistance, has listed CRE as being of critical priority because of their ability to transfer resistance genes, the severity of the infections and disease they cause, and their significant global burden (World Health Organization, 2024).

Ceftazidime-avibactam (CVA) is a unique combination of ceftazidime and the novel β-lactamase inhibitor avibactam, which inhibits the activities of Ambler classes A, C and partial D β-lactamase, including the *K. pneumoniae* carbapenemase (KPC) and OXA-48 (Zasowski et al., 2015). CVA was approved by the US Food and Drug Administration in 2015, and in China in 2019. It is indicated in China for the treatment of complicated intra-abdominal infections (cIAI) in adults and pediatric patients aged 3 months and older; hospital-acquired pneumonia (HAP), including ventilator-associated pneumonia (VAP); and infections with limited options caused by aerobic Gram-negative organisms in patients 18 years and older (Xu et al., 2023; Pfizer Ireland Pharmaceuticals, 2024; US Food and Drug Administration, 2025). The Infectious Diseases Society of America 2024 guidance on the treatment of antimicrobial-resistant Gram-negative infections recommends CVA for a range of infections caused by CRE (Tamma et al., 2024).

The efficacy of CVA has been assessed in phase 2 trials and in non-inferiority phase 3 trials conducted globally (Lucasti et al., 2013; Vazquez et al., 2012; Mazuski et al., 2016; Torres et al., 2018). Phase 2 and phase 3 trials comparing the efficacy of CVA plus metronidazole with meropenem in patients with cIAI, demonstrated that CVA was non-inferior for clinical cure rate for cIAI (Lucasti et al., 2013; Mazuski et al., 2016). Similarly, a comparison of the efficacy of CVA with meropenem in patients with HAP including VAP demonstrated non-inferiority of CVA in terms of clinical cure rate (Torres et al., 2018). In addition, a retrospective study in China evaluated the outcomes of 30 adult patients with multidrug-resistant Gram-negative bacterial infections. CVA was associated with a clinical response rate of 61.5% (16/26 patients) for infections including HAP, cIAI, bloodstream infections, urinary tract infections, and biliary duct infections, suggesting that this could be a suitable treatment option for such infections (Wang Q. et al., 2022). A real-world study examining the effectiveness of CVA versus polymyxin B in patients with carbapenem-resistant Gram-negative infections in western China noted clinical success, with lower rates of treatment failure at 28 days for CVA versus polymyxin B (Qu et al., 2023).

This observational study aimed to expand previous existing real-world evidence by describing the real-world usage, effectiveness, and antimicrobial features of CVA in clinical practice in China.

## Materials and methods

### Study design

This was a multicenter, prospective observational study with eligible patients enrolled across 15 clinical research centers in China (NCT05487586). Medical information was collected from patients’ medical records, and study outcomes were assessed and recorded in an electronic case report form (eCRF) by the investigators. Informed consent was obtained from all participants before starting the study. The final protocol, any amendments, and informed consent documentation were reviewed and approved by the Ethics Committee of Huashan Hospital, Fudan University, China (2022-687(C1)), and all 14 sites participating in the study. The informed consent document included the signature of the participant’s legal representative.

**Figure 1:**
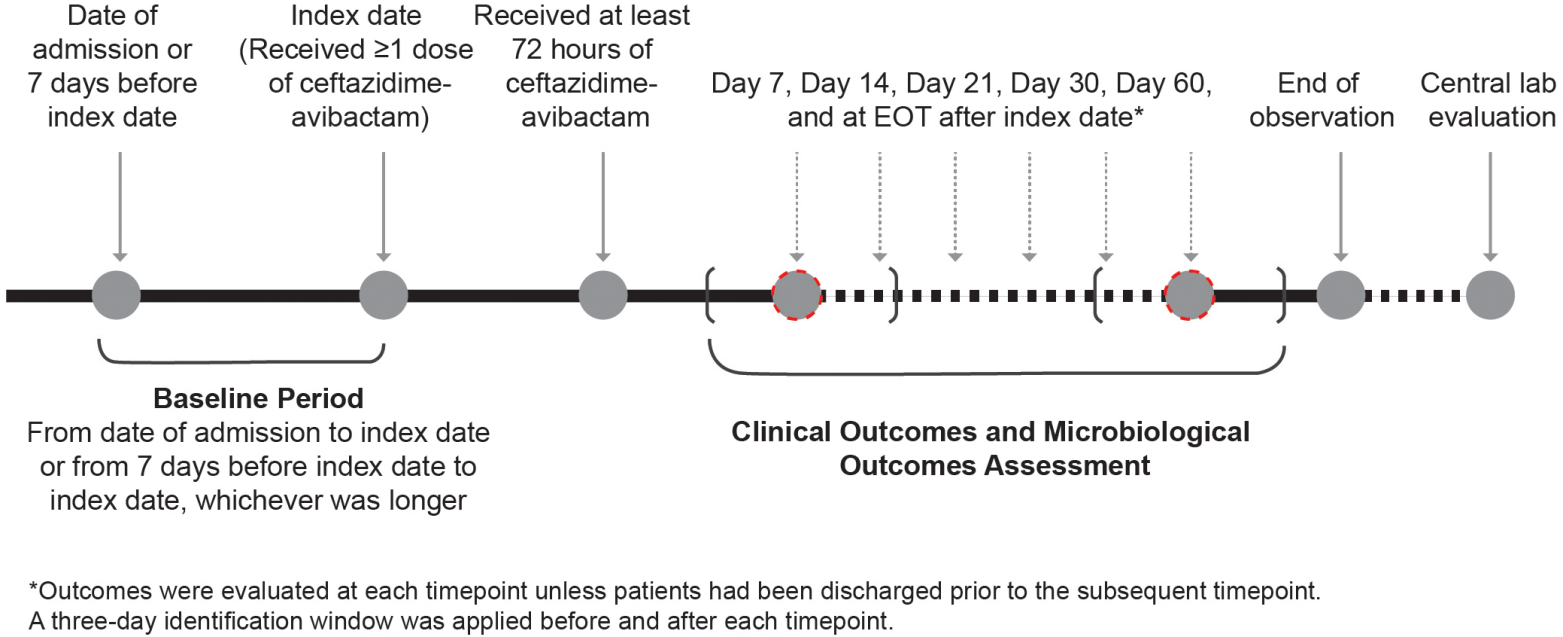
Study design.

The index date was defined as the date of initiation of ≥1 dose of CVA during the index hospitalization. The index hospitalization was defined as the patient’s first hospitalization that met the study eligibility criteria. The study baseline period was defined as either the time from date of admission until the index date, or the 7 days before the index date, whichever was longer. When patients had multiple records for variables of interest during the baseline period, the closest record to the index date was recorded. Patients were followed from the index date until death, withdrawal from the study, or 60 days following hospital discharge, whichever came first. Clinical outcomes and microbiological outcomes were observed until the end of treatment (EOT) according to clinical practice (**Figure 1**).

### Study population

Eligible patients were adult patients (≥18 years) who had been hospitalized and treated with ≥1 dose of CVA during hospitalization and provided signed informed consent. Each patient was only included in the study once using the first hospitalization that met the study eligibility criteria (**Supplementary Figure 1**). Patients participating in clinical trials, including non-interventional studies, were not eligible for inclusion in this study owing to the potential for protocol-driven activities that were not aligned with normal practice, and which could confound efficacy assessments; pregnant women were also excluded from this study.

### Analysis sets and outcome measures

The clinically evaluable (CE) analysis set included all enrolled patients with at least 72 hours use of CVA and ≥1 non-missing clinical evaluation outcome. The microbiologically evaluable (ME) analysis set comprised all enrolled patients with at least 72 hours use of CVA and ≥1 non-missing microbiological evaluation outcome (**Supplementary Table 1**).

Patient demographics and other characteristics were collected from patient medical records and described for all enrolled patients at baseline. These included: comorbidities, recent hospitalization, history of antibiotic exposure, pre-treatment disease severity, source of infection, and type of infection. The Deyo-Charlson Comorbidity Index (DCCI) was used to quantify comorbidity, as assessed by the investigator. Pre-treatment disease severity was measured at time of receiving first dose of CVA treatment via the Acute Physiology and Chronic Health Evaluation II (APACHE II) and Sequential Organ Failure Assessment (SOFA) score. Microbiological features of pathogens isolated at baseline including drug susceptibility were recorded. Hospital-acquired infections (HAI) were defined as infections that were typically not present or might not be incubating at the time of admission to hospital; these infections were usually acquired after hospitalization and manifested 48 hours after hospital admission. Community-acquired infections (CAI) were defined as those contracted outside of a hospital or might be incubating at the time of admission, diagnosed within 48 hours of hospital admission without any previous healthcare encounter. Limited Treatment Options (LTO) were defined as any infection with LTO, based upon clinical or microbiological evidence.

The primary outcomes were clinical cure rate, microbiological success rate, and real-world usage of CVA in clinical practice. Clinical cure rate was evaluated in the CE analysis set on Days 7, 14, 21, 30, and 60 and at EOT; it was measured as resolution of infection following treatment with CVA. Microbiological success rate was evaluated in the ME analysis set with microbiological samples collected at index date and on Days 7, 14, 21, 30, 60, and at EOT. Pre- and post-treatment microbiology samples and results were collected from laboratory records when available, along with collection of the microbiological outcome and the failure reason. The microbiological outcome and the failure reason were obtained from investigator assessment. Usage of CVA was evaluated in all enrolled patients, with the empiric or definitive usage of CVA recorded by investigators based on clinical practice and medical records. Empiric therapy was defined as therapy employed prior to release of microbiological test results (pathogen identified), whereas definitive therapy was defined as therapy given after release of microbiological test results (pathogen targeted).

Secondary study outcomes were evaluated and included description of antibiotic treatment administration; in-hospital length of stay; healthcare resource utilization; hospital readmission rate due to recurrence of infection in the same location 30- and 60-days post-discharge, including reason for readmission and date of readmission; and determination of in-hospital all-cause mortality.

### Statistical analyses

The actual number of enrolled patients was 228. All computations and generation of tables, listings, and data for figures were performed using SAS version 9.4 or higher. There were no statistical hypotheses and inference in this study. Descriptive statistics were applied. For interval estimate of proportion, a two-sided Clopper-Pearson exact confidence interval (CI) and a two-sided Wald CI was used. Unless otherwise specified in the description of the analyses, 95% CI was considered as a default (alpha = 5%) (**Supplementary Table 1**).

## Results

### Patient disposition

Overall, 228 patients were enrolled from 15 clinical research centers, with 220 (96.5%) patients meeting eligibility criteria and being included in the study (**Supplementary Figure 1**, **Supplementary Table 2**). Eight patients were excluded due to: lack of CVA administration during hospitalization (n = 3), being <18 years old at the time of informed consent (n = 2), not providing a signed informed consent form (ICF) (n = 2), and having been enrolled in another clinical trial (n = 1). For the majority of patients (n = 192, 87.3%), the end of observation occurred 60 days following hospital discharge. For the 28 patients who ended the observation before 60 days following hospital discharge, 22 patients (78.6%) died, and four (14.3%) were transferred to another hospital.

The CE analysis set consisted of 214 (97.3%) patients; the remaining 6 (2.7%) enrolled patients were not included due to duration of usage of CVA <72 hours. The ME analysis set included 208 (94.5%) patients; the remaining 12 (5.5%) patients were excluded due to missing all the ME outcomes (n = 9/12, 75.0%) and/or duration of CVA <72 hours (n = 6/12, 50.0%).

### Patient baseline demographics and characteristics

Two-thirds of patients were male (n = 150, 68.2%) and the mean (standard deviation [SD]) age was 62.4 (16.7) years (**Table 1**). Before the start of CVA treatment, the mean (SD) APACHE II score was 20.7 (8.4) and the mean (SD) SOFA score was 8.5 (4.5).

**Table 1:** Patient demographics and baseline characteristics.

Demographics and characteristics	All enrolled patients (N = 220)
Mean (SD) age, years	62.4 (16.7)
Male, n (%)	150 (68.2)
Baseline body mass index, mean (SD), kg/m^2^	22.5 (4.0)
Mean (SD) DCCI score	3.8 (3.3)
Hospitalized within 90 days prior to admission of current hospitalization, n (%)	147 (66.8)
Ward of admission for the current hospitalization, n (%)	
Neurosurgery	16 (7.3)
Respiratory	18 (8.2)
Urology	1 (0.5)
Oncology	0
Hematology	15 (6.8)
Infectious disease department	28 (12.7)
ICU	59 (26.8)
General surgery	15 (6.8)
Neurology	1 (0.5)
Other	66 (30.0)
Antibiotics used within 90 days prior to admission of current hospitalization	107 (48.6)
Infection susceptibility factors	
Glucocorticoid (≥10 mg Prednisone/day)	43 (19.5)
Immunosuppressive drugs	27 (12.3)
Hypoalbuminemia (<30 g/L)	115 (52.3)
Radiotherapy/chemotherapy	18 (8.2)
Leukopenia	9 (4.1)
Healthcare procedure within 30 days before CVA initiation	145 (65.9)
Pre-treatment disease severity, mean (SD)	
APACHE II score	20.7 (8.4)
SOFA score	8.5 (4.5)

APACHE II, Acute Physiology and Chronic Health Evaluation II; CVA, ceftazidime-avibactam; DCCI, Deyo-Charlson Comorbidity Index; ICU, intensive care unit; SOFA, Sequential organ failure assessment. N is the patient number for all enrolled patients. Percentages are based on all enrolled patients. Baseline body mass index = baseline weight (kg)/height (m)^2^.

### Prior antibiotic exposure

A total of 107 (48.6%) patients used antibiotics within 90 days prior to admission of their current hospitalization. The most frequently administered antibiotics were carbapenems (n = 46; 20.9%; primarily meropenem: n = 35, 15.9%), followed by β-lactamase inhibitor combinations including cefoperazone-sulbactam: n = 28, 12.7%; piperacillin-tazobactam: n = 24, 10.9%. Overall, 180 (81.8%) patients used antibiotics on or after admission and before CVA initiation for the current infection, with the most frequently administered antibiotics being carbapenems (n = 117; 53.2%; primarily meropenem: n = 85, 38.6%), followed by cefoperazone-sulbactam: n = 70, 31.8% and tigecycline: n = 53, 24.1%.

### Microbiological susceptibility at baseline

A total of 227 pathogens were identified among patients at baseline, the most commonly identified pathogens were *K. pneumoniae* (129 isolates), *P. aeruginosa* (33 isolates), *Serratia marcescens* (5 isolates), and *Enterobacter cloacae* (4 isolates). Of 87 K. *pneumoniae* isolates, 76 (87.4%) were meropenem resistant and the same proportion carried cabapenemase genes. Among the 76 K. *pneumoniae* isolates which produce carbapenemase, 73 carried blaKPC genes and three carried metallo-b-lactamase genes. Low levels of resistance to CVA were observed across all isolates (**Table 2**).

**Table 3:** Indication, type of infection, site and source of infection at index date (all enrolled patients).

n (%)	All enrolled patients (N = 220)
Indication for CVA at index date	
cIAI	37 (16.8)
HAP/VAP	129 (58.6)
LTO	49 (22.3)
LTO-specific infection [a]	
Bloodstream infection [b]	16 (32.7)
Pulmonary infection [c]	13 (26.5)
Urinary tract infection [d]	10 (20.4)
Biliary tract infection	3 (6.1)
Central nervous system infection	2 (4.1)
Other [e]	11 (22.4)
Source of infection	
HAI	134 (60.9)
CAI	40 (18.2)

CAI, community-acquired infection; cIAI, complicated intra-abdominal infections; CVA, ceftazidime-avibactam; HAI, hospital-acquired infection; HAP, hospital-acquired pneumonia; LTO, limited treatment options; VAP, ventilator-associated pneumonia.

N is the number of all enrolled patients.

Percentages are based on all enrolled patients, unless otherwise noted.

[a] Percentages are based on the number of patients with LTO.

[b] 1 case of bloodstream infection concurrent with pulmonary infection.

[c] 4 cases of pulmonary infection concurrent with infections at other sites.

[d] 2 cases of urinary tract infection concurrent with pulmonary infection.

[e] Including 2 COVID-19 associated pulmonary infection, 2 febrile neutropenia, 1 eye infection intraocular, 1 hepatitis, 1 infection, 1 infection prophylaxis, 1 liver abscess, 1 sinusitis, 1 soft tissue infection.

LTO-specific infections are coded using the MedDRA, version 25.1 (English version).

A patient having more than one LTO-specific infection within the same preferred term is counted only once.

### Site of infection at index date

Of the 220 patients, 142 (64.5%) had pneumonia, 37 (16.8%) had cIAI, and 16 (7.3%) had bloodstream infections at index date (**Table 3**). Infection type data at index were not available for five patients.

**Table 2:** Microbiological characteristics of first isolates.

Species, n	Meropenem resistance, n (% )	Produce carbapenemase		Ceftazidime-avibactam resistance, n (% )
		Serine β-lactamase, n (% )	Metallo-β-lactamase, n (% )	
*Klebsiella pneumoniae*, 87	76 (87.4%)	73 (83.9%)	3 (3.4%)	3 (3.4%)
*Pseudomonas aeruginosa*, 21	13 (61.9%)	0	0	2 (9.5%)
*Escherichia coli*, 4	2 (50.0%)	0	2 (50%)	2 (50.0%)
*Enterobacter cloacae*, 3	1 (33.3%)	0	1 (33.3%)	1 (33.3%)

### Clinical cure rate

**Figure 2:**
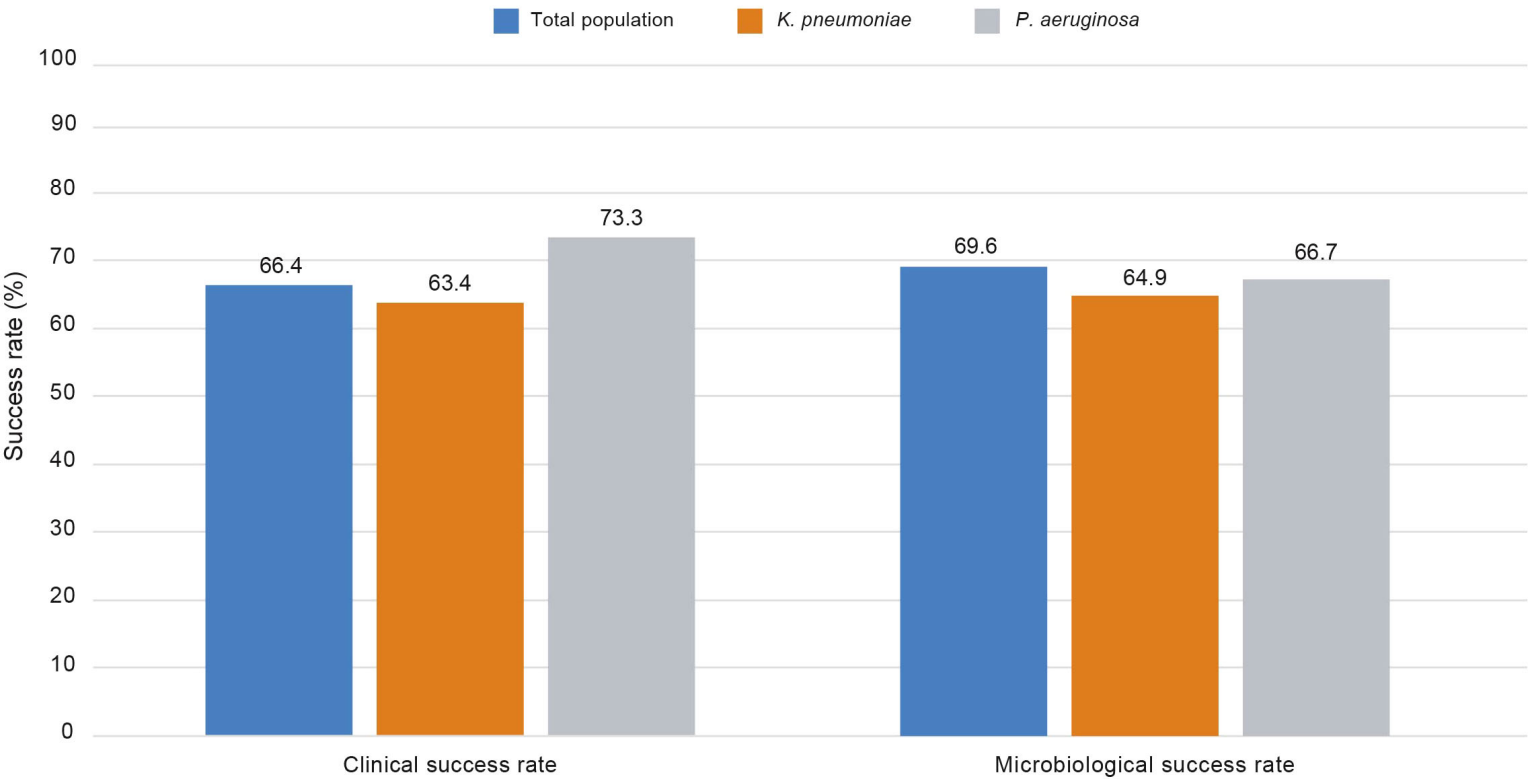
Clinical success rate (CE Analysis Set) and microbiological success rate (ME Analysis Set) at end of treatment, by pathogen. The clinical success rate is defined as: number of patients with clinical outcome “success” in a specific visit / number of patients with clinical outcome assessed in a specific visit. The microbiological success rate is defined as: number of patients with microbiological outcome “success” in a specific visit / number of patients with microbiological outcome assessed in a specific visit.

Clinical cure was achieved in 37.3% of patients at Day 7 (n = 57/153), 44.8% of patients at Day 14 (n = 56/125), 46.8% of patients at Day 21 (n = 44/94), 43.5% of patients at Day 30 (n = 30/69), and 69.0% of patients at Day 60 (n = 20/29). At EOT, clinical cure was achieved in 66.4% of patients (n = 142/214) overall (**Figure 2**). Clinical cure at EOT was achieved in 90.0% (n = 9/10) of patients with urinary tract infection (UTI), 68.8% (n = 11/16) of patients with bloodstream infection, 64.5% (n = 89/138) of patients with respiratory infections, and 61.1% (n = 22/36) of patients with cIAI (**Supplementary Figure 2**). There was only one patient with a central nervous system infection, and the clinical outcome for this patient was successful. The clinical cure rate was 63.4% for infections due to *K. pneumoniae* (n = 71/112) and 73.3% for *P. aeruginosa* (n = 22/30) at EOT.

### Microbiological success rate

Microbiological success was achieved in 69.6% of patients (n = 144/207) at EOT (**Figure 2**). Microbiological success was achieved in 80.0% (n = 8/10) of patients with UTI, 75.0% (n = 12/16) of patients with bloodstream infection, 69.9% (n = 93/133) of patients with respiratory infections, and 61.8% (n = 21/34) of patients with cIAI (**Supplementary Figure 1**). There was only one patient with a central nervous system infection, and the clinical outcome for this patient was unevaluable. The microbiological success rate was 64.9% for *K. pneumoniae* (n = 72/111) and 66.7% for *P. aeruginosa* (n = 20/30) at EOT. During the treatment process, two or more CRKP strains were isolated from 10 patients. In one case, resistance developed following 18 days of CVA treatment (the minimum inhibitory concentration [MIC] increased from 4 mg/L to >64 mg/L). Whole-genome sequencing analysis revealed that the carbapenemase mutation present had changed from KPC-2 to KPC-31.

### Real-world usage of CVA

Within the CE analysis set, 162 (75.7%) patients received definitive therapy of CVA and 52 (24.3%) received empiric therapy. The majority of patients (n = 200, 93.5%) received 2.5 g of CVA, mostly every 8 hours (q8h)(n = 173/200, 86.5%). The remaining patients (n = 14, 6.5%) received an adjusted dosage based on their renal function. A total of nine (4.2%) patients discontinued CVA treatment before the Day 60 visit, i.e., they did not complete treatment. Reasons for discontinuation included five patients due to treatment scheme modification/antibiotics downgraded, two patients due to infection being under control, one patient due to change of antibiotics, one patient due to extracted drug-resistant pathogen(s). No patients discontinued CVA treatment due to adverse events.

Mean (SD) duration of exposure to CVA was 13.7 (9. 9) days for patients in the CE analysis set. Overall, 172 (80.4%) patients received additional anti-infective therapy in combination with CVA, with the most frequently combined agents being tetracyclines (n = 76, 35.5%), polymyxins (n = 45, 21.0%), carbapenems (n = 40, 18.7%), third-generation cephalosporins (n = 27, 12.6%), aminoglycosides (n = 29, 13.6%) and others (n = 79, 36.9%).

### Hospitalization and healthcare resource utilization

Median (range) length of hospital stay (LOS) was 36.0 (6–751) days and median (min, max) LOS in the intensive care unit (ICU) was 25.0 (1–751) days. The median (min, max) length of mechanical ventilation was 17.0 (1,128) days. Twenty-two patients were readmitted to hospital due to a recurrence of infection in the same location within 30 days after discharge (n = 22/214, 0.10; Clopper-Pearson exact 95% CI: 0.07–0.15), and six patients were readmitted to hospital within 31 to 60 days after discharge (Clopper-Pearson exact 95% CI: 0.01–0.06). The proportion of patients with in-hospital all-cause mortality was 9.8% (n = 21/214, Clopper-Pearson exact 95% CI: 0.06–0.15).

## Discussion

This observational study assessed the real-world usage, effectiveness, and antimicrobial features of CVA in clinical practice in 220 adult patients in China. Infections were primarily pulmonary and hospital-acquired, with *K. pneumoniae* the most commonly identified pathogen, and the most common carbapenem-resistant pathogen. Among the *K. pneumoniae* isolates with carbapenemase gene data the majority carried *bla*_KPC_ genes. Three-quarters (75.7%) of patients received CVA as definitive therapy, with almost all patients receiving a dosage of 2.5 g q8h. Among the patients who received CVA for at least 72 hours, treatment success at EOT was achieved in 66.4% and was indeterminate in 8.9% of patients due to insufficient information to determine treatment success or failure. At EOT, microbiological success was achieved in 69.6% of patients.

Consistent with the findings from this study, CRE strains isolated in China have been shown to have a high prevalence of enzyme production, with the *bla*_KPC_ gene predominant among carbapenem-resistant *K. pneumoniae* (Han et al., 2020). The findings from the current study align with those from other studies, including a phase 3 multi-national double-blind randomized controlled trial (REPROVE; N = 817) which recruited patients from China and reported a non-inferior clinical cure rate of 68.8% at 28 days for patients with nosocomial pneumonia (including VAP) in the CVA group compared to 73.0% in the meropenem group (Torres et al., 2018). Notably, the REPROVE study also reported higher clinical cure rates in those with *K. pneumoniae* infections treated with CVA vs meropenem (83.8% vs 79.6%). Moreover, evidence from the literature suggests that CVA-based regimens have superior efficacy in patients with carbapenem-resistant infections compared with polymyxin B (Liu et al., 2025; Long et al., 2025).

In the recent real-world EZTEAM study, which was conducted across Europe and Latin America, as in the current study, *K. pneumoniae* was the most frequently identified pathogen (59.5% of patients in the EZTEAM study and 50.9% in the current study), with similar high comorbidity burden reported (DCCI score of 4.6 and 3.8 for EZTEAM and the current study, respectively). However, there were more patients starting CVA with an indication of HAP/VAP in the current study (58.6%) than in the EZTEAM study (22.1%) (Soriano et al., 2023). Respiratory infections were also the most common sources of infection recorded in a real-world study conducted in the US (Jorgensen et al., 2019). Of 203 patients, 43.8% of infections were caused by *K. pneumoniae*, and 117 patients had carbapenem-resistant infections (63.2% of these were *K. pneumoniae*). In another real-world study from France, OZAVIE, 34.2% of the 257 patients enrolled received ceftazidime-avibactam for the treatment of nosocomial pneumonia associated with infections principally caused by *Klebsiella* spp. (34.9%; with 47.2% of these producing extended spectrum β-lactamases) (Piroth et al., 2025). Findings from a recently published real-world study conducted in Taiwan similarly noted a high proportion of respiratory tract infections (46.2% of 472 patients) primarily due to infections with *K. pneumoniae* (64.4%), and a high proportion of patients with carbapenem-resistant isolates (348/472, 73.7%) (Yang et al., 2025). As in the current study, *P. aeruginosa* was often the second most commonly observed pathogen (13.4% in EZTEAM, 31.0% in the US study, 17.8% in the Taiwan study and 17.7% in the current study), but was the principal pathogen isolated in OZAVIE (52.4%) (Soriano et al., 2023; Jorgensen et al., 2019; Yang et al., 2025; Piroth et al., 2025). Multidrug-resistant *P. aeruginosa* isolates were also observed (69.2% in EZTEAM, 17.6% in OZAVIE) (Piroth et al., 2025; Soriano et al., 2023). In most of these studies, clinical success was similar to that noted in the current study (77.3% in EZTEAM, in the US study 70.9% of patients experienced either clinical success or an indeterminate outcome, 79.0% in OZAVIE), but was lower in the Taiwan study (58.1%) (Soriano et al., 2023; Jorgensen et al., 2019; Yang et al., 2025; Piroth et al., 2025).

The observed microbiological outcomes also align with those from a phase 2 randomized study in hospitalized adults with serious complicated urinary tract infections (N = 135) due to Gram-negative pathogens. In this study, a favorable microbiological response was achieved in 70.4% of patients receiving CVA compared to 71.4% of patients receiving imipenem-cilastatin at 5–9 days post-therapy (Vazquez et al., 2012). Similarly, in the phase 3 REPRISE study, per-patient microbiological response rates at test of cure were higher with CVA (118/144 [81.9%; 95% CI, 75.1, 87.6]) than with best available therapy (88/137 [64.2%; 95% CI, 56.0, 71.9]) (Carmeli et al., 2016). Real-world studies have also demonstrated the microbiological effectiveness of CVA (Zhuang et al., 2025; Todi et al., 2024). Clinical and microbiological success rates reported by a retrospective electronic health records-based study were 76.3% and 60.3%, respectively at EOT with CVA (Todi et al., 2024).

Comparisons between CVA and polymyxins, which are commonly used for the treatment of carbapenem-resistant infections in China, suggest that CVA may be associated with lower all-cause hospital mortality and higher treatment success and bacterial clearance in patients with carbapenem-resistant Gram-negative infections (van Duin et al., 2018; Chen et al., 2022, Yang W. et al., 2023). Findings from a propensity score-matched multicenter real-world study to compare CVA with polymyxin B for the treatment of carbapenem-resistant *K. pneumoniae* infections demonstrated significantly higher rates of both clinical efficacy (71.3% vs. 56.1%; p = 0.011) and microbiological clearance (74.7% vs. 41.4%; p < 0.001) (Zhuang et al., 2025). Taken together, the findings from the current study add to the body of real-world evidence that CVA is an effective treatment for carbapenem-resistant organisms in China and provide insights into real-world usage.

The current study demonstrated several strengths. It is one of the few real-world studies to focus on the clinical routine use of CVA in a Chinese patient population. Compared to restrictive eligibility criteria in randomized controlled trials, this study reflects routine clinical practice in China by allowing selection of a heterogeneous patient population with various comorbidities and concomitant treatments across representative tertiary hospitals. Additionally, most of the real-world studies on CVA in China were retrospective studies. In contrast, the current study was designed to follow patients prospectively, which limited the restrictions that normally derive from the nature of the retrospective study design. Moreover, prospective studies typically require informed consent, which better protects patient rights. Prospective studies also allow for the preservation of isolated bacterial strains, enabling future analysis of the molecular characteristics of these strains, such as their carbapenemase enzyme types.

Notwithstanding, there are some limitations that should be considered when interpreting the results. Patient enrollment was lower than expected, primarily owing to a high use of generic products and recruitment challenges during the COVID-19 pandemic. As a single-arm observational study, comparative analyses were not possible. The centers participating in the study were tertiary hospitals, which may limit the generalizability of these findings given that the patient population may not be representative of other hospitals in terms of comorbidities, indications, and severity of infection. Depending on the routine practice, data such as microbiological features of isolated strains and clinical and microbiological outcomes might not be systematically measured or available. In addition, only a limited number of microbiological samples could be assessed by a central laboratory due to issues with sample delivery from clinical centers, which limits the interpretation on microbiological outcome assessed by central laboratory and only a proportion of patients enrolled had genetic analyses of the infecting pathogen. An additional limitation of this study is the lack of long-term outcome data beyond 60 days after hospital discharge, which would have allowed a more comprehensive evaluation of treatment outcomes. It might be beneficial for future studies to include longer-term follow-up to allow a fuller evaluation of the effect of the study treatments on patient outcomes.

## Conclusion

The findings from this study provide important real-world evidence on treatment patterns, microbiological features, and clinical and microbiological outcomes for CVA in routine clinical practice in China. This large-scale, multicenter study yields results similar to those of previously published clinical trials, supporting the clinical use of CVA in China, especially in the treatment of resistant infections, including those caused by carbapenem-resistant *K. pneumoniae*. The study includes multiple infection sites and provides reference data for infections at specific sites. Clinical and microbiological outcomes from this study were comparable with those reported in the clinical trials and previous real-world studies, providing real-world evidence in support of the favorable benefit of CVA treatment in China. Findings from this study complement the results of randomized controlled trials and offer insights to assess and improve clinical practice in China.

## Data Availability

The original contributions presented in the study are included in the article/**Supplementary Material**. Further inquiries can be directed to the corresponding author.
