# Supplementary material for: Real-world usage, effectiveness, and microbiological features of ceftazidime-avibactam in clinical practice in China
Source: Front Cell Infect Microbiol. 2026 Jan 27;15:1663392. doi: 10.3389/fcimb.2025.1663392 (PMC12886437; doi:10.3389/fcimb.2025.1663392)
Supplement: Supplementary file 1 [file DataSheet1.docx]

Supplementary Material

### Supplementary Table 1. Summary of Statistical Analyses

| Statistical Analysis | Analysis Set | Statistical Method |
| --- | --- | --- |
| Disposition and withdrawals | All enrolled patients | Descriptive statistics |
| Analysis sets | All enrolled patients | Descriptive statistics |
| Primary endpoints | | |
| Demographics and other baseline characteristics | All enrolled patients | Descriptive statistics |
| Microbiological results at baseline | All enrolled patients | Descriptive statistics |
| Clinical success rate | CE analysis set | 1. Point estimation 2. Interval estimation:   Wald CI, Clopper-Pearson exact CI |
| Microbiological success rate | ME analysis set | 1. Point estimation 2. Interval estimation:   Wald CI, Clopper-Pearson exact CI |
| Microbiological success rate by pathogen | ME analysis set | 1. Point estimation 2. Interval estimation:   Wald CI, Clopper-Pearson exact CI |
| Secondary endpoints | | |
| Concomitant procedures | All enrolled patients; CE analysis set | Descriptive statistics |
| Length of mechanical ventilation | All enrolled patients; CE analysis set | Descriptive statistics |
| Study medication exposure | All enrolled patients; CE analysis set | Descriptive statistics |
| Combination therapy with ceftazidime-avibactam | All enrolled patients; CE analysis set | Descriptive statistics |
| LOS in-hospital | All enrolled patients; CE analysis set | Descriptive statistics |
| LOS in ICU | All enrolled patients; CE analysis set | Descriptive statistics |
| Admission and discharge diagnosis | All enrolled patients; CE analysis set | Descriptive statistics |
| Readmission | All enrolled patients; CE analysis set | 1. Point estimation 2. Interval estimation:   Wald CI, Clopper-Pearson exact CI |
| In-hospital all-cause mortality | All enrolled patients; CE analysis set | 1. Point estimation 2. Interval estimation:   Wald CI, Clopper-Pearson exact CI |

CE, clinically evaluable; CI, confidence interval; ICU, intensive care unit; LOS, length of stay; ME, microbiologically evaluable.

### Supplementary Table 2. Analysis Sets (All Enrolled Patients)

|  | All Enrolled Patients (N = 228) |
| --- | --- |
|  |  |
| All enrolled patients [a] | 220 (96.5) |
|  |  |
| Clinically evaluable analysis set [b] | 214 (97.3) |
| Not included in the clinically evaluable analysis set | 6 (2.7) |
| Reasons not included in the clinically evaluable analysis set [d] |  |
| Usage of CVA <72 hours | 6 (100.0) |
| Missed all clinical evaluation outcomes | 0 |
|  |  |
| Microbiologically evaluable analysis set [c] | 208 (94.5) |
| Not included in the microbiologically evaluable analysis set | 12 (5.5) |
| Reasons not included in the microbiologically evaluable analysis set [e] |  |
| Usage of CVA <72 hours | 6 (50.0) |
| Missed all microbiological evaluation outcomes | 9 (75.0) |

CVA, ceftazidime-avibactam.

[a] All enrolled patients include all eligible patients. The eligible patients must meet the inclusion criteria and must not meet the exclusion criteria.

[b] The clinically evaluable analysis set includes all enrolled patients with at least 72 hours use of CVA and at least one non-missing clinical evaluation outcome. Percentages are based on all enrolled patients.

[c] The microbiologically evaluable analysis set includes all enrolled patients with at least 72 hours use of CVA and at least one non-missing microbiological evaluation outcome. Percentages are based on all enrolled patients.

[d] Percentages are based on the number of all enrolled patients but not included in the clinically evaluable analysis sets.

[e] Percentages are based on the number of all enrolled patients but not included in the microbiologically evaluable analysis sets.

N is the number of all enrolled patients. Percentages are based on the number of all enrolled patients, unless otherwise noted. If a patient has more than one reason not to be included in the clinically evaluable analysis set, all reasons are counted in the subcategories under "Reasons not included in the clinically evaluable analysis set", but that patient is counted only once in the category "Not included in the clinically evaluable analysis set". If a patient has more than one reason not included in the microbiologically evaluable analysis set, all reasons are counted in the subcategories under "Reasons not included in the microbiologically evaluable analysis set", but that patient is counted only once in the category "Not included in the microbiologically evaluable analysis set".

### Supplementary Figure 1. Patient disposition

**
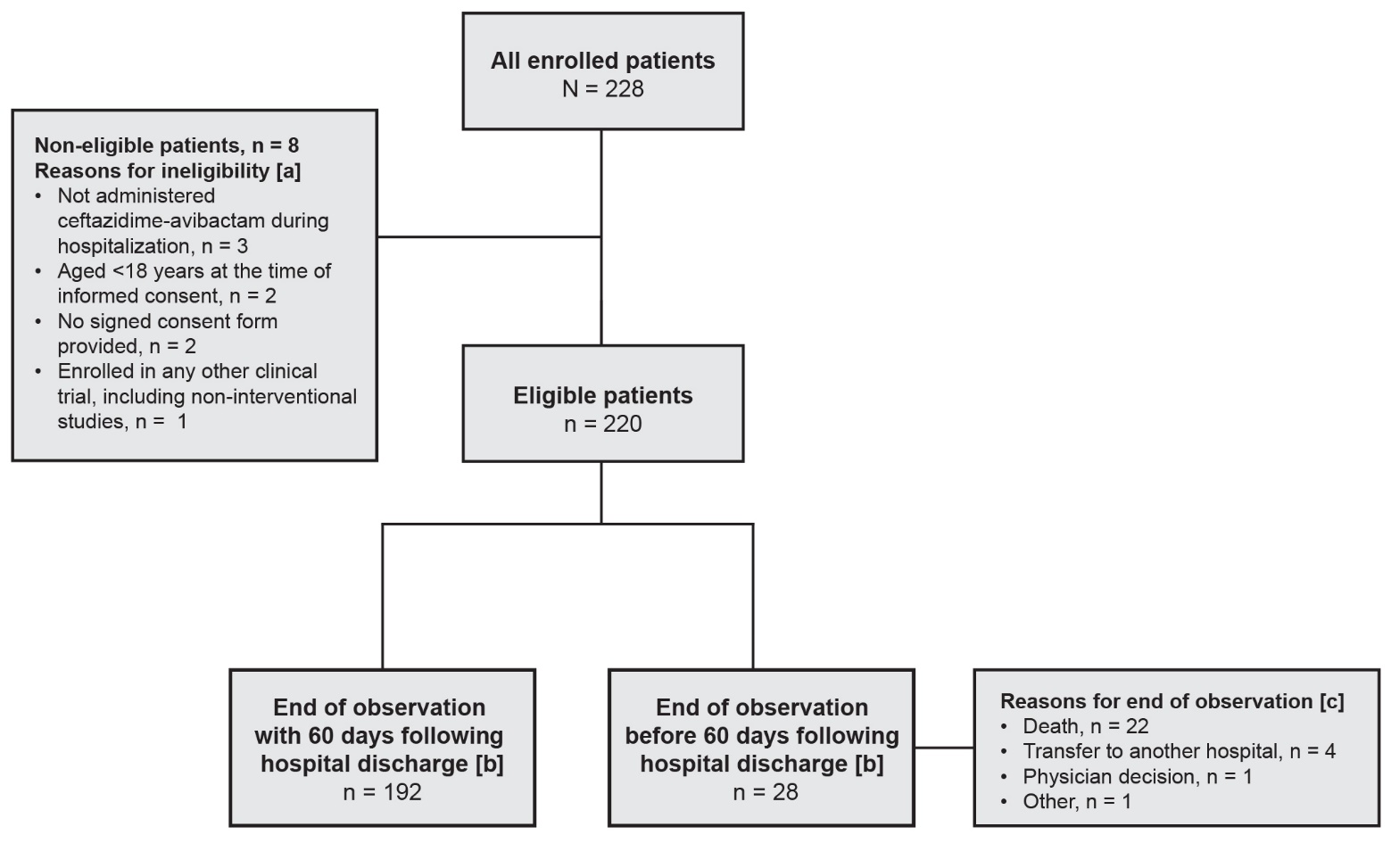
**

N is the number of enrolled patients.

Percentages are based on the number of all enrolled patients, unless otherwise noted.

[a] Percentages are based on the number of non-eligible patients.

[b] Percentage is based on the number of eligible patients who meet the inclusion criteria and do not meet the exclusion criteria.

[c] Percentages are based on the number of patients with end of observation before 60 days following hospital discharge.

**Supplementary Figure 2.** Clinical and microbiological outcome at EOT for infection sites (with at least 10 cases)

BSI, blood-stream infection; cIAI, complicated intra-abdominal infection; EOT, end of treatment; UTI, urinary tract infection.
